# Injuries to Aboriginal populations living on- and off-reserve in metropolitan and non-metropolitan areas in British Columbia, Canada: Incidence and trends, 1986-2010

**DOI:** 10.1186/s12889-016-3078-x

**Published:** 2016-05-13

**Authors:** Mariana Brussoni, M. Anne George, Andrew Jin, Christopher E. Lalonde, Rod McCormick

**Affiliations:** Department of Pediatrics, School of Population & Public Health, British Columbia Injury Research & Prevention Unit, Child and Family Research Institute, University of British Columbia, F511 – 4480 Oak Street, Vancouver, British Columbia V6H 3V4 Canada; Department of Pediatrics, School of Population & Public Health, Child and Family Research Institute, University of British Columbia, F508 – 4480 Oak Street, Vancouver, British Columbia V2N 3Z9 Canada; Epidemiology Consultant, 2762 – 133 Street, Surrey, British Columbia V4P 1X9 Canada; Department of Psychology, University of Victoria, PO Box 1700, Victoria, British Columbia V8W 2Y2 Canada; Faculty of Human, Social and Educational Development, Thompson Rivers University, 900 McGill Road, Kamloops, British Columbia V2C 0C8 Canada

**Keywords:** First Nations, Indigenous population, Indians, Accidents, Population surveillance, Socioeconomic factors

## Abstract

**Background:**

Disparities in injury rates between Aboriginal and non-Aboriginal populations in British Columbia (BC) are well established. Information regarding the influence of residence on disparities is scarce. We sought to fill these gaps by examining hospitalization rates for all injuries, unintentional injuries and intentional injuries across 24 years among i) Aboriginal and total populations; ii) populations living in metropolitan and non-metropolitan areas; and iii) Aboriginal populations living on- and off-reserve.

**Methods:**

We used data spanning 1986 through 2010 from BC’s universal health care insurance plan, linked to vital statistics databases. Aboriginal people were identified by insurance premium group and birth and death record notations, and their residence was determined by postal code. “On-reserve” residence was established by postal code areas associated with an Indian reserve or settlement. Health Service Delivery Areas (HSDAs) were classified as “metropolitan” if they contained a population of at least 100,000 with a density of 400 or more people per square kilometre. We calculated the crude hospitalization incidence rate and the Standardized Relative Risk (SRR) of hospitalization due to injury standardizing by gender, 5-year age group, and HSDA. We assessed cumulative change in SRR over time as the relative change between the first and last years of the observation period.

**Results:**

Aboriginal metropolitan populations living off-reserve had the lowest SRR of injury (2.0), but this was 2.3 times greater than the general British Columbia metropolitan population (0.86). For intentional injuries, Aboriginal populations living on-reserve in non-metropolitan areas were at 5.9 times greater risk than the total BC population. In general, the largest injury disparities were evident for Aboriginal non-metropolitan populations living on-reserve (SRR 3.0); 2.5 times greater than the general BC non-metropolitan population (1.2). Time trends indicated decreasing disparities, with Aboriginal non-metropolitan populations experiencing the largest declines in injury rates.

**Conclusions:**

Metropolitan/non-metropolitan residence appears to be a more important predictor than on/off-reserve residence for all injuries and unintentional injuries, and the relationship was even more pronounced for intentional injuries. The persistent disparities highlight the need for culturally sensitive and geographically relevant injury prevention approaches.

## Background

Disparities in injury rates when comparing Aboriginal and non-Aboriginal populations in many countries with indigenous populations are well documented [1–6]. In Canada, the Aboriginal population includes First Nations (North American Indians), Inuit and Métis people. A recent Canadian study found that injury hospitalization rates were highest in geographic areas with the greatest percentage of individuals identifying as First Nations (146/10,000 person years), followed by Métis (112), and Inuit (100) [7]. In contrast, injury rates in areas with a low percentage of individuals identifying as Aboriginal were substantially lower (55/10,000 person years). Our previous research in British Columbia (BC), the Canadian province with the largest number of Aboriginal bands, indicated encouraging downward injury trends and a narrowing gap between Aboriginal and total populations [8]. However, disparities persist, along with efforts to understand root causes and develop culturally and locally relevant prevention strategies.

Canada’s Aboriginal population reflects a vast array of cultures, linguistic and geographic circumstances [9, 10]. Over 1.4 million Canadians identified as Aboriginal in 2011, representing 4.3 % of the total population [11]. In BC, over 232,000 people identified as Aboriginal, representing 16.6 % of the Canadian Aboriginal population, and 5.4 % of the total BC population. There are 155,020 self-identified First Nations people in BC, of which 73 % are registered as having Indian Status, as defined by the Indian Act of Canada [11].

Historical colonial practices and policies, such as banning Aboriginal traditional practices and removing children from their families for residential school education, aimed to decimate Aboriginal cultural heritage and subjugate Aboriginal populations to White European rule [10, 12]. Colonial policies of environmental dispossession included restricted access to traditional territories and physical displacement and forced migration of Aboriginal communities onto ‘Lands Reserved for Indians,’ (a.k.a., reserves) per Section 91.24 of Canada’s Constitution Act. These policies have had profound and far-reaching effects on the health and wellbeing of Aboriginal communities in Canada and the fabric of their cultural continuity [10, 13]. Some argue that policies of environmental dispossession, in particular, are among the most culturally harmful and stressful because of the intimate interrelationship between Aboriginal culture and traditional lands [12, 14]. Many Aboriginal communities lost access to the physical and ecological features of their traditional environments, could no longer continue their cultural practices, eat traditional foods, nor use traditional medicines [12]. Furthermore, Canadian history – past and present – is replete with examples of environmental compromising and contamination of Aboriginal lands, which has further eroded traditional culture, subsistence and lifestyles [15–17].

Duran and Duran highlight the psychological effects of colonial legacies: loss of power, despair, and self-hatred [18]. These have obvious implications for mental health, physical health and injuries, such as externalization of self-hatred through violence toward others, or internalization through violence toward self (e.g., suicide, self-harm) [19]. Furthermore, these effects may not only manifest through intentional injuries, but more subtly as a diminished concern for personal safety, which can influence personal protective behaviours (e.g., not using seat belts, reckless driving, substance use). The resulting injuries (e.g., motor vehicle crashes, poisoning) would be coded as unintentional in administrative datasets.

The critical population health approach considers the processes and drivers that influence the social determinants of health and seeks to deconstruct the social, economic and ideological forces that undermine health for given populations; in so doing, promoting social, economic and political forces for emancipation and health promotion [12, 20]. Given the legacy of colonial injustices perpetrated on Aboriginal populations in Canada, the critical population health approach is essential to any study of this issue and can be used to understand and promote conditions that ease disparities and promote equity among all populations.

### RISC research project

While it is well established that Aboriginal populations have higher rates of injury than non-Aboriginal populations, these population-level statistics mask considerable variability between different Aboriginal communities; which can illuminate potential paths for prevention. A case in point is Chandler and Lalonde’s analysis of youth suicide rates among First Nations communities in BC, which showed that some bands experienced many times the rate of youth suicides than others [21–23]. Their work illustrated the importance of cultural continuity in combating the powerful and destructive legacy of colonial policies. They reported a negative relationship between the extent to which bands had managed to maintain cultural continuity – measured by markers such as band control over their cultural heritage, health, education, political structures, and land claims – and youth suicide rates.

Our RISC (Reducing Injury: Surveillance, Culture) research project [19], was inspired by Chandler and Lalonde’s work and guided by the critical population health approach to broaden the analysis beyond suicides to explore other intentional injuries, such as assaults, as well as unintentional injuries, such as work-related injuries and falls [8, 24, 25]. We accessed BC population-level linked datasets that enabled unprecedented analyses of Aboriginal people’s injury-related hospitalization rates, primary care visits and worker compensation injury claims. We hypothesized that there would be broad diversity in injury rates for different Aboriginal communities, as illustrated in Chandler and Lalonde’s suicide work [21, 22]. Our hypothesis was based on the following assumptions: (a) the colonial legacy would manifest physically across injury types (not just suicide); and (b) there would be differences across Aboriginal communities in social determinants of health, such as geographic living conditions, education levels and socioeconomic conditions.

Our work to date has supported our hypothesis and assumptions. In a previous paper, we reported results indicating vast differences in several social determinants of health across Aboriginal communities, including levels of income, education, housing quality, work participation and conditions, and geographic circumstances [26]. For example, while some communities had high school graduation rates of 100 %, others had 0 %. Similarly, employment rates ranged from 8 to 77 %. As expected, differences were also reflected in injury-related hospitalizations, with some communities having rates four times lower than the total BC population, while others were at over nine times greater risk. We examined which community-level factors were significant risk markers for injury-related hospitalization, and found that only lower proportion of high school graduates, and greater geographic remoteness remained significant in multivariable analyses [26].

In this paper, we use a critical population health approach to further explore the influence of residence as a determinant of injuries for Aboriginal populations in BC. Aboriginal populations in BC can live in any of the following places: metropolitan on-reserve, non-metropolitan on-reserve, metropolitan off-reserve and non-metropolitan off-reserve. As outlined in the sections below, each of these residences has different geographic and cultural implications that can potentially influence injuries.

### Rural residence

Regardless of Aboriginal status, geographic remoteness influences disparities in injury rates among Canadians. Injury-related death rates are up to 79 % higher in rural areas compared to metropolitan areas, and disparities widen with increasing rurality [27]. Behavioural and environmental factors influence disparity in injury rates between metropolitan and rural areas. Rural residents have lower perceptions of risky behaviours and are less likely to use safety devices [28, 29]. Many of the most hazardous occupations (e.g., mining, forestry, agriculture) are based in rural areas, and the rural environment can be more dangerous with hazards such as roads with fewer safety features [29–32]. Recreational activities common in rural areas, such as the use of all-terrain vehicles, snowmobiles, and boats, also represent a leading cause of injury mortality [27, 33]. Past research suggests that Aboriginal and non-Aboriginal populations in rural communities engage in similar risk-taking behaviours, suggesting that many of these behaviours relate to living in rural environments, rather than Aboriginal culture *per se* [34, 35].

We would expect that Canada’s Aboriginal populations would disproportionately experience the injury-related disadvantages associated with rural living because they are more likely to live in rural areas (46.8 %) [36], than non-Aboriginal Canadians (20 %) [37]. In BC, the Northern Health Authority is the largest and least populous of BC’s five geographic health authorities, yet includes the largest proportion of Aboriginal people (16.6 %). This compares to the Fraser and Vancouver Coastal Health Authorities, which comprise metropolitan Vancouver, and have the lowest proportion of Aboriginal population at 2.5 and 2.3 %, respectively [38]. Similar to Canada, Australian Aboriginal populations are also more likely to live in rural areas and research there has confirmed that the elevated motor vehicle injury rates found in Aboriginal populations relate to rural living [39].

Effects of colonial traumatisation may also be evident for Aboriginal populations living in rural areas. Place [40] presented Canadian data comparing Aboriginal and non-Aboriginal rural populations that indicate disparities in social determinants of health: Aboriginal populations living in rural areas were less likely to have high school certificates (21.5 % versus 25.2 %) and had unemployment rates more than double those of non-Aboriginal rural populations (14.4 % versus 6.7 %). Our previous research has found that lower levels of education attainment was positively associated with general injury rates [26], and lower labour force participation was positively associated with intentional injury rates [41].

### Reserve residence

An increasing proportion of Aboriginal people are living off-reserve and in urban areas [42]. Among BC’s Status First Nations population in 2008, 52.6 % were living off-reserve, of which 37.6 % were in the three most populous health authorities [38].

Tjepkema [43] and Place [40] present data that suggest a trend toward better health for off-reserve populations, but with considerable variability depending on the health behaviour in question (e.g., smoking, physical activity, alcohol consumption). A lack of significance testing in either report limits interpretations of the findings. Furthermore, the confounding effect of socioeconomic factors renders relationships difficult to untangle.

Socioeconomic factors are generally worse for on-reserve than off-reserve residents. For example, among BC’s Aboriginal on-reserve population, 19.8 % had a high school certificate and 75.8 % participated in the labour force, compared to 26.2 and 84.9 %, respectively for the off-reserve population [44]. What effect these factors have on health in general, and injuries in particular, is not clear and likely influenced by the profound diversity evident in different Aboriginal communities.

While socioeconomic indicators may point to improved circumstances for off-reserve residents, the “group density effect” would predict better health for on-reserve populations [45]. This effect occurs when the positive psychological and health impacts associated with residing in more advantaged areas are overridden by the psychological stress and stigma of being treated as a low status minority group by the majority community. The ongoing and systemic racism experienced by Canada’s Aboriginal population may result in a “group density effect” whereby living on-reserve and surrounded by others similar in cultural, level of isolation and socioeconomic background as oneself can render psychological and health-related advantages.

### Research questions

In this paper, we sought to examine the influence of residence on injury-related hospitalization rates for all injuries, unintentional and intentional injuries among i) Aboriginal and total populations; ii) populations living in metropolitan and non-metropolitan areas; and iii) Aboriginal populations living on- and off-reserve. We also analyzed injury rate time trends across 24 years of data. In defining metropolitan and non-metropolitan, we adapted the Statistics Canada definition, referring to “large population centres” (population of at least 100,000 with a density of 400 or more people per square kilometre) as “metropolitan” areas and all others as “non-metropolitan” areas [46].

## Methods

### Ethics review and permission for data access

The University of British Columbia Behavioural Research Ethics Board reviewed and approved our methods. The Data Stewards representing the BC Ministry of Health Services and the Vital Statistics Agency of BC approved the data access requests. We used existing databases, permanently linked by British Columbia Personal Health Number, maintained by Population Data BC. Population Data BC rendered the client records anonymous before our analysis. **Disclaimer**: all inferences, opinions, and conclusions drawn in this journal manuscript are those of the authors, and do not reflect the opinions or policies of the Data Stewards.

### Population counts

We obtained annual snapshots of the consolidated registration and premium billing files of the Medical Services Plan of BC (the universal provincial health care insurance program), representing fiscal years 1985-86 through 2010-2011 [47]. We took this to represent the total resident population of BC. Within this population, we marked as “Aboriginal” any person with:

- Membership in Medical Services Plan Premium Group 21 (insurance premiums paid by First Nations and Inuit Health Program, Health Canada, for reason of Aboriginal status); or
- One or both parents with Aboriginal status or resident on an Indian Reserve, as indicated on the linked Vital Statistics birth record [48]; or
- Aboriginal status or resident of a First Nation reserve, as indicated on the linked Vital Statistics death record [49].

We previously described this method, and discussed the quality of the population registry, and validity and limitations of the Aboriginal identification [8, 24–26].

We classified as “on-reserve” those Aboriginal people residing in a postal code area associated with an Indian reserve or settlement recognized by Statistics Canada and the federal Department of Aboriginal Affairs and Northern Development. We classified all other Aboriginal people as “off-reserve”.

There are sixteen Health Service Delivery Areas (HSDAs) in BC [50]. We classified these as “metropolitan” (HSDAs 22, 23, 31, 32, 33 and 41, comprising metropolitan Vancouver and metropolitan Victoria) or “not metropolitan” (HSDAs 11, 12, 13, 14, 21, 42, 43, 51, 52, and 53). Vancouver and Victoria are the two largest Census Metropolitan Areas in BC, containing 60.4 % of the population enumerated in BC by the 2011 Census of Canada [51]. The categories of “metropolitan” area and “non-metropolitan” area are respectively the same as the categories we called “urban” and “not urban” in our previous reports [8, 24, 25].

### Hospitalization counts

We tabulated discharge summary records representing hospital separations occurring in BC from January 1, 1986 through March 31, 2010 [52]. We considered hospitalization as “due to injury” if the level of care was “acute” or “rehabilitation,” and the Most Responsible Diagnosis on the discharge record was an International Classification of Diseases Revision 9 (ICD-9) numeric code in the range 800 through 999, or an International Classification of Diseases Revision 10 (ICD-10) code in the range S00 through T98 (describing type of injury and area of the body affected). From April 1, 1991 on, hospitalizations were also classified by intention and external cause, based on the first occurrence of a supplemental injury diagnosis code (ICD-9 codes E800 through E999, or ICD-10 codes V01 through Y98). For purposes of this analysis, we classified hospital separations occurring in BC from April 1, 1991 through March 31, 2010 as “unintentional” (ICD-9 E800-E928, E930-E949, or ICD-10 V01-X59, Y40-Y84) or “intentional” (ICD-9 E950-E958, E960-E968, or ICD-10 X60-Y09). Linking discharge records to the population registry, we tabulated counts of hospitalizations by year and the patient’s age, gender, HSDA of residence, Aboriginal status, and reserve residence. Non-residents of BC were excluded from the hospitalization counts.

### Risk of injury

For the Aboriginal population of BC (categorized by metropolitan or non-metropolitan, and on-reserve or off-reserve residence), and the total population of BC (categorized by metropolitan or non-metropolitan residence), and for unintentional injuries, intentional injuries, and all injuries, we calculated the crude rate of hospitalization due to injury per 10,000 person-years, Standardized Relative Risk (SRR) of hospitalization due to injury (relative to the total population of BC and standardizing by gender, 5-year age group, and HSDA), and 95 % confidence intervals of the crude rate and SRR, using the method of indirect standardization [53]. We tested the statistical significance of the difference between two SRRs by calculating the probability (2-sided, z-test) that Ln((SRR_2_)/(SRR_1_)) = zero.

Cumulative change in SRR was assessed over time as the relative change between the first and last years of the observation period, i.e., (SRR_2_/SRR_1_) - 1. To facilitate comparisons, we converted relative change over a period of multiple years to an annualized change, using the following formula.

$$ {\left(\frac{SR{R}_2}{SR{R}_1}\right)}^{1/\left({t}_2-{t}_1\right)}-1 $$

We compared the cumulative change (from the first to the last years) among Aboriginal people to the cumulative change over the same period among the total population of BC. We tested the statistical significance of the difference by calculating the probability (2-sided, z-test) that Ln((SRR_2_)/(SRR_1_)) Aboriginal = Ln((SRR_2_)/(SRR_1_)) BC.

## Results

As seen in Table 1, most of the BC population lives in metropolitan areas, however the Aboriginal population largely dwells in non-metropolitan areas. Approximately half (54 %) of BC’s Aboriginal population lives off-reserve. While the off-reserve population is more likely to live in metropolitan areas than the on-reserve population, there are a greater proportion of Aboriginal populations living in non-metropolitan areas, whether on- or off-reserve.

**Table 1:** Mean annual population count by residence in BC, 1986-2010

	BC Population		Aboriginal Populations					
			Total		Off-reserve		On-reserve	
	N	%	N	%	N	%	N	%
Metropolitan	2,339,054	60.9	36,697	29.3	26,025	38.4	10,672	18.6
Non-metropolitan	1,504,740	39.1	88,637	70.7	41,804	61.6	46,833	81.4
Total	3,843,794	100	125,334	100	67,829	100	57,505	100

Table 2 shows injury hospitalization rates per 10,000 for the different populations of BC. The BC population residing in metropolitan areas has the lowest total, unintentional and intentional injuries rates. Aboriginal populations living on-reserve in non-metropolitan areas had the highest rates of injury, regardless of intentionality. Among Aboriginal populations, those living off-reserve in metropolitan areas have the lowest total and unintentional injury rates. Aboriginal populations living on-reserve in metropolitan areas had the lowest intentional injury rates.

**Table 2:** Crude incidence of hospitalization due to injury, British Columbia, 1991-2010

Population	Total injuries			Unintentional			Intentional		
	Rate^a^	95 % CI		Rate^a^	95 % CI		Rate^a^	95 % CI	
		From	To		From	To		From	To
BC Total	91	91	91	83	82	83	8	8	9
BC metropolitan	77	77	78	70	70	70	7	7	7
BC non-metropolitan	112	112	113	102	101	102	11	10	11
Aboriginal Total	215	213	217	140	139	142	45	44	46
Aboriginal, on-reserve metropolitan	207	201	212	148	143	154	37	34	40
Aboriginal, on-reserve non-metropolitan	263	260	266	171	168	174	51	50	52
Aboriginal, off-reserve metropolitan	158	155	161	103	100	105	40	38	41
Aboriginal, off-reserve non-metropolitan	199	197	202	129	126	131	45	43	46

^a^Crude rate per 10,000 person years

Table 3 shows the SRR of injury-related hospitalizations for all injuries. As expected, the BC population residing in metropolitan areas is at the lowest risk of injury, followed by those dwelling in non-metropolitan areas. Of the Aboriginal populations, those living off-reserve in metropolitan areas have the lowest SRR of all injuries (1.99), but their risk of injury is 2.3 times greater risk than the BC metropolitan population (0.86). The non-metropolitan Aboriginal population living on-reserve – the group with the highest SRR of injury hospitalization (3.04) – is at 2.5 times greater risk of injury than the non-metropolitan-dwelling BC population (1.24). The differences comparing the Aboriginal and total BC populations for both metropolitan and non-metropolitan areas are statistically significant. The patterns for unintentional injuries are the same, with the SRR being several times higher for Aboriginal than total BC populations. Aboriginal populations living on-reserve in non-metropolitan areas are at 5.9 times greater risk of intentional injuries than the total BC population. Among Aboriginal populations, the lowest SRR of intentional injury is evident among those living on-reserve in metropolitan areas.

**Table 3:** Standardized Relative Risk of hospitalization due to injury, British Columbia, 1991-2010

Population	Total injuries			Unintentional			Intentional		
	SRR^a^	95 % CI		SRR^a^	95 % CI		SRR^a^	95 % CI	
		From	To		From	To		From	To
BC Total	1.0	[reference]		1.0	[reference]		1.0	[reference]	
BC metropolitan	0.86	0.85	0.86	0.86	0.85	0.86	0.84	0.83	0.84
BC non-metropolitan	1.24	1.23	1.24	1.23	1.23	1.24	1.30	1.28	1.31
Aboriginal Total	2.61	2.58	2.64	2.19	2.16	2.23	5.25	5.04	5.48
Aboriginal, on-reserve metropolitan	2.44	2.34	2.54	2.24	2.13	2.36	4.22	3.69	4.93
Aboriginal, on-reserve non-metropolitan	3.04	2.98	3.10	2.53	2.47	2.59	5.85	5.47	6.29
Aboriginal, off-reserve metropolitan	1.99	1.93	2.04	1.68	1.63	1.74	4.58	4.19	5.03
Aboriginal, off-reserve non-metropolitan	2.51	2.46	2.56	2.10	2.04	2.16	5.29	4.93	5.71

^a^Risk relative to total population of BC, standardized by age and gender

The differences in the category of intentional injuries, between on-reserve and off-reserve Aboriginal populations, when stratified by metropolitan or non-metropolitan residence, are not statistically significant: on-reserve non-metropolitan SRR = 5.85 vs. off-reserve non-metropolitan SRR = 5.29, *p* = 0.0502 (2-sided), and on-reserve metropolitan SRR = 4.22 vs. off-reserve metropolitan SRR = 4.58, *p* = 0.3472 (2-sided). On the other hand, the differences between metropolitan and non-metropolitan Aboriginal populations, when stratified by on- or off-reserve residence, are statistically significant: on-reserve non-metropolitan SRR = 5.85 vs. on-reserve metropolitan SRR = 4.22, *p* < 0.0001 (2-sided), and off-reserve non-metropolitan SRR = 5.29 vs. off-reserve metropolitan SRR = 4.58, *p* = 0.0141 (2-sided).

Table 4 shows changes in SRR from 1986 to 2010 for geographic and reserve residence. Trends indicate that among all British Columbians, the risk of injury-related hospitalization decreased over the 24 years. In addition, the percent decreases are greater for the Aboriginal population than for the total BC population, which means that the disparities between the Aboriginal and total BC populations are decreasing. Aboriginal non-metropolitan populations experienced the largest drop (69.1 %) in their SRR of injury hospitalization.

**Table 4:** Change in SRR of hospitalization due to injury by residence in BC, 1986-2010

Population	SRR		% change	p*	Annual % change		
	1986	2010				95 % CI	
						From	To
BC Total^a^	1.42	0.67	-52.6 %	NA	-3.1 %	-3.1 %	-3.0 %
BC, metropolitan^a^	1.19	0.59	-50.5 %	NA	-2.9 %	-3.0 %	-2.8 %
BC, non-metropolitan^a^	1.84	0.79	-56.9 %	NA	-3.4 %	-3.6 %	-3.3 %
Aboriginal Total^a^	3.36	1.18	-64.8 %	0.000	-4.3 %	-4.7 %	-3.8 %
Aboriginal, metropolitan^a^	3.15	1.49	-52.8 %	0.703	-3.1 %	-4.1 %	-2.1 %
Aboriginal, non-metropolitan^a^	4.75	1.47	-69.1 %	0.000	-4.8 %	-5.4 %	-4.2 %
Aboriginal, Off-reserve^b^	3.12	1.13	-63.9 %	0.001	-4.2 %	-4.8 %	-3.5 %
Aboriginal, On-reserve^b^	3.50	1.26	-64.1 %	0.001	-4.2 %	-4.8 %	-3.5 %

^a^SRR standardized by age and gender, relative to total population of BC, 1986 to 2010

^b^SRR standardized by age, gender and Health Service Delivery Area, relative to total population of BC, 1986 to 2010

*probability (2-sided, z-test) that Ln((SRR 2010)/(SRR 1986)) Aboriginal = Ln((SRR 2010)/(SRR 1986)) BC total

## Discussion

The largest proportion of BC’s Aboriginal populations lived on-reserve in non-metropolitan areas (37.3 %). While previous research indicated that the Canadian off-reserve population was mostly urban [40, 54], in BC the majority of the off-reserve population lived in non-metropolitan areas (61.6 %). The differences in findings may relate to differences regarding the definition of urban and rural. Our definition of non-metropolitan area included small cities such as Prince George (population: 72,000), which other research might classify as urban. We used Statistics Canada’s definition of “large population centre” to divide our sample [55].

Our findings comparing all injury-related hospitalizations indicate that (a) non-metropolitan residents are at significantly greater risk than metropolitan residents; (b) Aboriginal populations are at significantly greater risk than total populations; (c) Among Aboriginal populations, on-reserve non-metropolitan populations are at greatest risk, followed by off-reserve non-metropolitan, on-reserve metropolitan, and off-reserve metropolitan. The pattern of Aboriginal populations being at higher risk than the total BC population remained consistent for all injuries, unintentional injuries or intentional injuries, with Aboriginal on-reserve non-metropolitan populations having SRRs 2.5, 2.1 and 5.3 times higher, respectively.

Our findings suggest independent and interaction effects of metropolitan/non-metropolitan area and on/off-reserve residence. Living in non-metropolitan areas resulted in a greater SRR of injury hospitalization, regardless of injury intentionality (SRRs = 2.10-5.85) than living in metropolitan areas (SRRs = 1.68-4.58), whether on- or off-reserve. Our findings suggest that metropolitan/non-metropolitan residence appears to be a more important predictor than on/off-reserve residence for all injuries, unintentional injuries and intentional injuries. This relationship was even more pronounced for intentional injuries. Our analysis of differences in SRR of intentional injury stratified by either metropolitan or reserve residence indicated that the difference between metropolitan and non-metropolitan residence remained significant, while on- and off-reserve residence did not.

One possibility for the relative importance of metropolitan/non-metropolitan over reserve residence relates to the larger non-metropolitan Aboriginal population in BC, relative to the Aboriginal metropolitan population. Having a greater proportion of Aboriginal populations may dilute the group density effect in on-reserve non-metropolitan areas. In metropolitan areas, the result may be related to the greater availability of ambulatory care options. Lower hospitalization rates have been observed for all causes of diseases in more urban areas, where other options, such as outpatient and home care, are more readily accessible [56].

With respect to intentional injuries and the lack of protective effect of living off-reserve, it is possible that different factors related to on- or off-reserve residence influence intentional compared to unintentional injuries. For example, while opportunities for education, labour force participation, and access to specialist health care tend to be better off-reserve, there may also be more experiences of racism, exclusion and marginalization [54]. These can enhance the group density effect, as well as promote negative feelings that can manifest in explicit intent to harm oneself or another [18, 19].

The fact that BC’s Aboriginal population – whether dwelling in metropolitan or non-metropolitan areas – experiences significantly more injury-related hospitalizations than the total population, highlights the fact that enduring differences in health equity transcend geographic location. The disparities exist for both unintentional and intentional injuries, but are particularly pronounced for intentional injuries, with SRRs indicating more than four times greater risk. This may suggest that while the colonial legacy manifests across injury types, it is more pronounced for intentional injuries, which, as discussed above, involve a more conscious choice to harm oneself or another person. Further research is needed to untangle the competing effects of socioeconomic status, geographic residence, and Aboriginal status as risk factors for injury.

Time trends indicate that injury-related hospitalizations have decreased by at least 50 % for everyone in BC, but that the biggest drop is evident for Aboriginal populations living in non-metropolitan areas (69 %), indicating a decrease in injury disparities. This may result from improvements in injury prevention measures, particularly in non-metropolitan areas. A number of measures to reduce motor vehicle crashes – the primary source of injury disparity between urban and rural areas – have been implemented during the time span covered by this study [57]. Some notable road safety benchmarks include: the requirement for daytime running lights in 1990; graduated licensing programs implemented in most jurisdictions between 1994-2005; in 2008, changes to the Canadian Criminal Code improved detection of impaired driving; and BC enacted booster seat laws in 2008 [57].

In previous publications, we have outlined the limitations associated with the data and our methods [8, 24–26]. We would also add that time trend data may reflect differential reporting of injury among population groups over time. For instance, previous research indicates that Indigenous people have significantly lower rates of health care access and utilization compared to non-Indigenous people [58]. Specific interventions to address the barriers that disproportionately affect them would, by design, differentially influence the injury rates. In addition, readers may wish to apply their own correction to the stated p-values to account for multiple comparisons.

## Conclusions

This study outlines significant disparities in injury-related hospitalizations that exist for Aboriginal populations in BC. In addition to being at significantly greater risk than the total population, Aboriginal people face differential risks depending on where they reside, with on-reserve non-metropolitan populations at highest risk. While time trends across 1986 to 2010 that indicate decreasing injury disparities among Aboriginal populations are encouraging, the persistent disparity highlights the need for ongoing efforts to identify culturally sensitive and geographically relevant injury prevention approaches to narrow the gap.

### Ethics

The University of British Columbia Behavioural Research Ethics Board reviewed and approved our methods.

### Consent for publication

Not applicable.

### Availability of data and materials

The data that we studied [47–49, 52] are available on request, from Population Data BC (https://www.popdata.bc.ca/data, contact: Kelly Sanderson, Researcher Liaison Unit Lead, email: kelly.sanderson@popdata.bc.ca; specifications in project file George 11–012) subject to approval by the Data Stewards representing the British Columbia Ministry of Health Services, and the Vital Statistics Agency of British Columbia, for ethical and privacy reasons, because the data pertain to individuals. The data may be accessed and statistically analysed only on Population Data BC’s Secure Research Environment cloud server.

## Abbreviations

BC: British Columbia
HSDA: Health Service Delivery Area
ICD: International Classification of Diseases
RISC: Reducing Injury: Surveillance, Culture
